# Biostimulants and herbicides a tool to reduce non-commercial yield tubers and improve potato yield structure

**DOI:** 10.1038/s41598-023-47831-0

**Published:** 2023-11-22

**Authors:** Agnieszka Ginter, Krystyna Zarzecka, Marek Gugała, Iwona Mystkowska

**Affiliations:** 1 Faculty of Agricultural Sciences, University of Siedlce, Prusa Street 14, 08-110 Siedlce, Poland; 2 Department of Dieteties, John Paul II University of Applied Sciences, Sidorska Street 95/97, 21-500 Biała Podlaska, Poland

**Keywords:** Ecology, Plant sciences

## Abstract

The basis for the study was a field experiment conducted in 2012–2014 in the production fields of multi-branch Soleks company in Wojnów, the district of Siedlce in eastern Poland. The experiment was established in a split-plot arrangement as a two-factor experiment in three replications. The first factor were: three cultivars of edible potato—Bartek, Gawin, Honorata, and the second factor were: five objects of potato cultivation with herbicides and biostimulants: 1—Control object—without chemical protection, 2—herbicide Harrier 295 ZC, 3—herbicide Harrier 295 ZC + biostimulant Kelpak SL, 4—herbicide Sencor 70 WG, 5—herbicide Sencor 70 WG + biostimulant Asahi SL. The aim of the study was to reduce the non-commercial potato yield and improve the yield structure through the application of biostimulants and herbicides, and to determine the relationship between weed infestation and tuber yield. The least amount of weeds and the best destruction efficiency were obtained after the application of herbicide Sencor 70 WG + biostimulant Asahi SL and herbicide Harrier 295 ZC + biostimulant Kelpak SL. Effective reduction of weed infestation contributed to improvement of yield structure and reduction of potato non-commercial yield. Based on correlation coefficients, a significant relationship between weed infestation and potato non-commercial yield was shown.

## Introduction

Modern agriculture aims to reduce industrial inputs (chemical pesticides, mineral fertilizers), while aiming to increase productivity and obtain high-quality raw materials. One way to do this is to provide plants during vegetation with good conditions for growth and development using biostimulants^1,2^. The use of seaweed extracts and humic substances as plant growth stimulators, also referred to as biostimulants, has been on the rise for several, several years. Formulations containing seaweed extracts and humic acids promote plant growth, increase tolerance to abiotic stresses, and increase the efficiency of nutrient utilization by plants^3^. In addition, they increase yields and improve quality traits, increase resistance to many diseases^4–6^. Potato is the world's fourth crop and the main human food after rice, wheat and corn, hence it is important to obtain high yields with good nutritional value^7^. Biostimulants used in the cultivation of this crop increase yield and content of tuber components, alleviate the effects of stress, and reduce disease infestation^5,8–11^. On the other hand, biostimulants applied together with herbicides are more effective in reducing weed infestation in plantations, improving yield structure and chemical composition of tubers, and increasing the profitability of cultivation^12–14^. However, research on the combined use of herbicides and biostimulants in potato cultivation is scarce. Hence, the research undertaken was aimed at reducing the non-commercial yield of potato and improving the tuber yield structure through the use of biostimulants and herbicides. In this study, the research hypothesis was that herbicides with biostimulants would effectively reduce weed infestation, thereby improving the yield structure and reducing the non-commercial yield of potato.

## Methods

### Research location and soil conditions

The results of the study come from a three-year field experiment conducted in 2012–2014 in production fields of multi-branch Soleks company in Wojnów, the district of Siedlce, in Mazovia Voivodeship, in Poland (52° 12′ 59″ N, 22° 34′ 37″ E). The research was conducted on soil classified according to the World Reference Base for Soil Resources^15^ belonging to Haplic Luvisol (LV-ha) with a slightly acid reaction (pH in KCl 5.60–6.35). The content of available forms of macronutrients in soil level 0–30 cm in mg kg^−1^ was as follows: phosphorus high to very high (68.6–110.0), potassium medium to very high (129.0–149.4) and magnesium high (50.0-56.0). The soil was analysed at the Chemical and Agricultural Station in Wesoła, near Warsaw, in Poland. The soil analysis are in accordance with the tables of Chemical and Agricultural Station in Poland.

### The factors of the experiment

The field experiment was set up by split-plot design in triplicate as a two-factor experiment. The factors of the experiment were:

I three edible potato cultivars—Bartek, Gawin, Honorata (Table 1).
II five potato treatment objects with herbicides and biostimulants (Table 2):
1 control object—without chemical protection,
2 herbicide Harrier 295 ZC (linuron and clomazone)—single spraying, 1.5 l ha^−1^, BBCH 00-08,
3 herbicide Harrier 295 ZC (linuron and clomazone)—single spraying 1,5 l ha^−1^, BBCH 00-08 + biostimulant Kelpak SL—double spraying, 1.0 l ha^−1^, BBCH 13–19 and 0.5 l ha^−1^, BBCH 31–35,
4 herbicide Sencor 70 WG (metribuzin)—single spraying, 1.5 l ha^−1^, BBCH 00-08,
5 - herbicide Sencor 70 WG (metribuzin)—single spraying 1.5 l ha^−1^, BBCH 00-08 + biostimulant Asahi SL—double spraying, 1.0 l ha^−1^, BBCH 13–19 and 0.5 l ha^−1^, BBCH 31–35.

**Table 1:** Factor I—selected characteristics of potato cultivars^16^.

Characteristic	Cultivars		
	Bartek	Gawin	Honorata
Breeder	Zamarte-Poland	Strzekęcin-Poland	Europlant-Germany
Maturity time	Medium early	Medium early	Medium early
Total yield t ha^−1^	54,4	44.7	44.1
Tuber size scale 1–9	7.0	7.0	7.0
Starch mg kg^−1^ FM	161	165	156
Flesh color	Light yellow	Light yellow	Light yellow
Taste scale 1–9	6.9	6.4	6.7
Vitamin C mg kg^−1^ FM	246	127	228

**Table 2:** Factors II—characteristics of objects in the field research.

No.	Objects	Characteristic
1	Control object	Mechanical weeding only, without chemical protection
2	Harrier 295 ZC dose 2.0 dm3 ha-1	Herbicide (linuron + clomazone)
3	Harrier 295 ZC dose 2.0 dm^3^ ha^−1^ + Kelpak SL dose 2.0 dm^3^ ha^−1^*	Herbicide—(linuron + clomazone) and biostimulant contains: Corg—0.36%, org. substance—32.9%, extract from Brown algae *Ecklonia maxima* (11 mg dm^−3^ auxins and 0.031 mg dm^−3^ cytokines, which means a 350:1 auxin to cytokine ratio. Producer—Kelp Products (Pty) Ltd., P.O. Box 325, Simon’s Town, the Republic of South Africa
4	Sencor 70 WG dose 1 kg ha^−1^	Herbicide (metribuzin)
5	Sencor 70 WG dose 1 kg ha^−1^ + Asahi SL dose 1.0 dm^3^ ha^−1^*	Herbicide (metribuzin) and biostimulant contains: natural nitropherols found in plants: sodium ortho-nitropherol 0.2%, sodium para-nitropherol 0.3%, sodium 5-nitroguaiacol 0.1%. Producer—Asahi Chemical Europe s.r.o., Lužná 591/4—Vokovice, 160 00 Praha 6, Republika Czech Republic

*The chemical composition of biostimulants is given according to the Institute of Soil Science and Plant Cultivation^17^. 1—Control object; 2—Harrier 295 ZC; 3—Harrier 295 ZC + Kelpak SL; 4—Sencor 70 WG; 5—Sencor 70 WG + Asahi SL.

The timing of the treatments, as well as tuber planting and potato harvesting, is shown in Table 3.

**Table 3:** Treatments carried out in the field experiment.

Treatments	Objects		Years	
		2012	2013	2014
Potato tuber planting time	1–5	30.04.2012	08.05.2013	23.04.2014
Ridding of potato rows	1,4,5	05.05.2012	13.05.2013	27.04.2014
Ridding with harrowing	1,4,5	10.05.2012	18.05.2013	02.05.2014
Spraying herbicide Harrier 295 ZC (linuron + clomazone)	2,3	10.05.2012	18.05.2013	02.05.2014
Ridding of potato rows	1,4,5	22.05.2012	29.05.2013	18.05.2014
Spraying herbicide Sencor 70 WG (metribuzin)	4,5	22.05.2012	29.05.2013	18.05.2014
Spraying biostimulant Kelpak SL (*Ecklonia maxima*)	3	06.06. and 20.06.2012	12.06. and 24.06.2013	31.05. and 20.06.2014
Spraying biostimulant Asahi SL (sodium ortho-nitrophenol, sodium para-nitrophenol, sodium 5-nitroguaiacol)	5	06.06. and 20.06.2012	12.06. and 24.06.2013	31.05. and 20.06.2014
Ridding of rows after emergence of potato plants	1	07.06.2012	12.06.2013	10.06.2012
Ridding of rows after emergence of potato plants	1	12.06.2012	19.06.2013	19.06.2014
Date of harvest	1–5	04.09.2012	04.09.2013	02.09.2014

1—Control object; 2—Harrier 295 ZC; 3—Harrier 295 ZC + Kelpak SL; 4—Sencor 70 WG; 5—Sencor 70 WG + Asahi SL.

The field experiment included 45 plots. The area of one experimental plot was 18.73 m^2^ = 5.55 m × 3.375 m, i.e. 15 plants every 37 cm × 5 ridges every 67.5 cm = 75 plants.

### Fertilization and chemical protection

In each year of the study, the forecrop was winter wheat. All plots in the experiment were fertilized with the same dose of manure and mineral fertilizers. In autumn, manure was applied at a rate of 25.0 t ha^−1^ and phosphorus and potassium fertilizers at rates of—44.0 kg ha^−1^ P (46% triple superphosphate) and 124.5 kg ha^−1^ K (60% potassium salt), which were covered with pre-winter plowing. Nitrogen was applied in the spring, before planting the tubers, at a rate of 100 kg ha^−1^ N (34% ammonium salt). The fertilizer was introduced into the soil at a depth of 15–20 cm. Potato protection against diseases and pests was applied according to the recommendations of the Institute of Plant Protection—National Research Institute^18^. During the growing seasons, fungicides were used against the potato blight: Ridomil Gold MZ 68 WG (mancozeb and metalaxyl-M) and Altima 500 SC (fluazinam). Colorado potato beetle was inspected by using the insecticides: Apacz 50 WG (clothianidin) and Fastac 100 EC (alpha and cypermethrin).

### Determination of weed infestation, non-commercial yield and structure tubers

Analysis of weed infestation during potato vegetation was carried out by the quantitative-weight method twice: before potato row closing (phase BBCH 34-35)^19^ and before harvest of the potato (phase BBCH 97). The BBCH-scale is a system for a uniform coding of phenologically similar growth stages of all mono—and dicotyledonous plant species. The abbreviation BBCH derivers from Biologische Bundesanstalt, Bundessortenamt and Chemical industry. The scale is used in the European Union countries to make characteristic of the development phases of plants. Weeds were determined in an area of 1.0 m^2^, defined by a 33.4 × 150 cm (5010 cm^2^) frame. The frame was randomly thrown at three consecutive locations in the plot diagonally through the ridges. Weed control efficacy was expressed as a percentage of weed number destruction relative to a control plot tended only mechanically, according to the methodology of Badowski^19^. Each year, just before tuber harvest, 10 plants from each plot were randomly dug up. The number and weight of tubers with diameters < 35, 36–50, 51–60 and > 60 mm were determined. Non-commercial and commercial yield was determined. The weight of tubers of the < 35 mm diameter fraction and the weight of tubers with external and internal defects present in the other sample fractions (greened tubers, tubers damaged by soil pests, severely deformed tubers, disease-infested tubers and severely physiologically cracked tubers) were taken as the non-commercial yield of tubers^20^.

### Statistical analysis

The results of weed evaluation—total number of weeds determined before the rows were short-circuited and before the tuber was harvested, and the non-commercial yield of potato were subjected to analysis of variance. The significance of the sources of variation was tested with the Fischler-Snedecor 'F' test, and the evaluation of the significance of differences at a significance level of P ≤ 0.05 between the compared averages was performed using Tukey's multiple intervals^21^. The relationship between weed infestation and non-commercial tuber yield was determined using linear correlation coefficients. All calculations were made in Excel 2016 using the authors’ algorithm by using the mathematical model:

$${\text{Yijl }} = {\text{ m}} + {\text{ai}} + {\text{gl}} + {\text{e}}/{1}/{\text{il}} + {\text{bj}} + {\text{abij}} + {\text{e}}/{2}/{\text{ijl}},$$

### Indications in the model

Yijl means value of characteristic researched: I means the level of A (cultivars) j means the level of B (cultivars) in the first replication, m means the experimental average, ai means the effect of i-level of A (cultivars), gl means the first replication effect, e/1/il means the random effect of a (cultivars) with replications, bj means the effect of j-level of B (objects), abij means the interaction effect of A (cultivars) and B (objects), e/2/ijl means random error.

### Weather conditions

**Figure 1:**
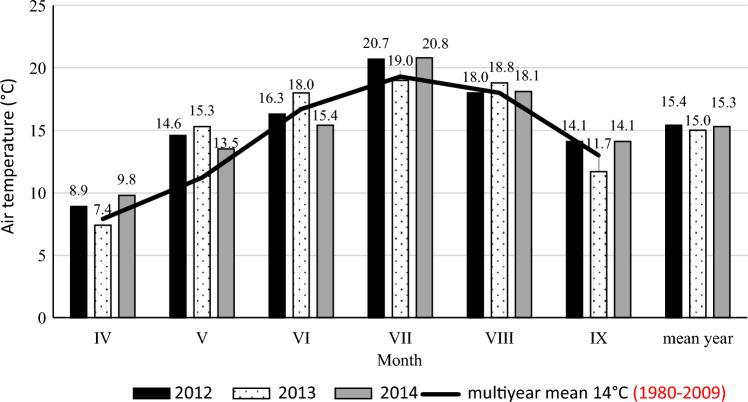
Air temperature during the vegetative growth periods of potato (Zawady Meteorogical Station in Poland).

**Figure 2:**
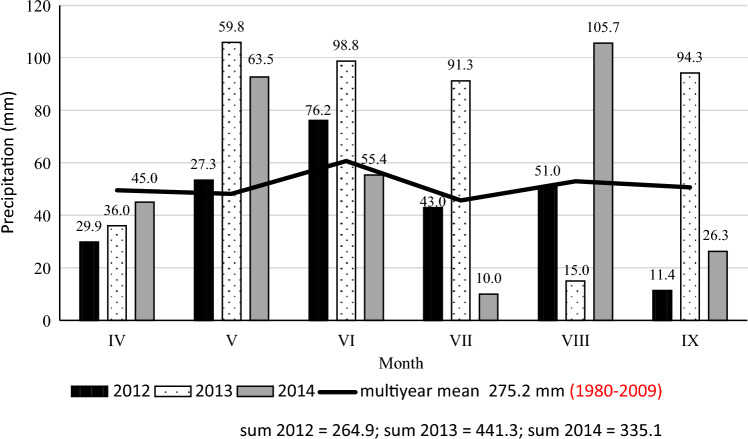
Precipitation during the vegetative growth periods of potato (Zawady Meteorogical Station in Poland).

The course of weather conditions in the years of the study differed significantly from the air temperature and precipitation of the 1980–2009 multi-year period (Table 4, Figs. 1 and 2). The Meteorological Station in Zawady is located about 8.0 km from the experimental field.

**Table 4:** Sielianinov hydrothermal coefficient and description of the months in the 2012–2014 growing seasons^22^.

Month	Years		
	2012	2013	2014
	Hydrothermal coefficient and description of the month (K)**		
April	1.10—relatively dry	1.60—optimal	1.50—optimal
May	1.20—relatively dry	2.30—humid	2.30— humid
June	1.60—optimal	1.80—relatively humid	1.20—relatively dry
July	0.70—very dry	1.60—optimal	0.16—extremely dry
August	0.90—dry	0.30—extremely dry	1.90—relatively humid
September	0.27—extremely dry	2.70—very humid	0.62—very dry
April-September	0.95—dry	1.60—optimal	1.20—relatively dry

**Description of the month was calculated according to formula: K = 10 P/ Σt, where: P—the sum of the monthly rainfalls in mm, Σt—monthly total air temperature > 0 °C. Ranges of values are classified as follows: up to 0.4: extremely dry; 0.41–0.7: very dry; 0.71–1.0: dry; 1.01–1.3: relatively dry; 1.31–1.6: optimal; 1.61–2.0: relatively humid; 2.01–2.5: humid; 2.51–3.0: very humid; over 3.0: extremely humid.

In 2012, precipitation was lower than in the multi-year period, and temperatures were higher; it was a dry year (K = 0.95). The months of July and August, which determine tuber formation and yield accumulation, were very dry (K = 0.70) and dry (K = 0.90). The year 2013 was warmer and more abundant in precipitation (K = 1.60—optimal) than in the perennial period, which was favorable for potato yield, and the non-commercial yield was the lowest. In 2014, precipitation was unevenly distributed, July was extremely dry (K = 0.16), and the growing season was relatively dry (K=1.20).

### Ethical approval

All methods of experimental research and field studies on cultivated plants, including the collection of plant material were carried out with the relevant institutional, national guidelines and legislation.

## Results and discussion

### Weed number and weed efficiency control

Potato yield and quality are determined by many factors, mainly agrotechnical treatments, variety, soil or moisture-thermal conditions during vegetation^11,23–25^. One of the most important factors limiting yield is careless cultivation and the presence of weeds. In the conducted studies, the number of weeds per unit area, determined at the beginning and at the end of the growing season, depended significantly on the methods of care and on weather conditions during the growing seasons (Tables 5 and 6).

**Table 5:** Number of weeds at the beginning and end of potato vegetation and non-commercial yield of potato.

Objects	Cultivars			Mean	Efficiency of weed control (% of control object)
	Bartek	Gawin	Honorata		
Number of weeds per 1 m^2^ before potato row closing					
1	12.6	14.2	16,1	14.3	–
2	4.6	5.7	6.1	5.5	61.5
3	2.9	3.0	3.6	3.1	78.3
4	3.5	4.3	5.8	4.6	67.8
5	1.9	2.0	2.3	2.1	85.3
Mean	5.1	5.9	6.8	5.9	73.2
* LSD*_0.05_ for: cultivars—ns; objects—1.4; interaction: cultivars × objects—ns					
Number of weeds per 1 m^2^ before harvest of the potato					
1	9.4	12.6	11.0	11.0	–
2	7.1	9.3	7.5	8.0	27.2
3	2.0	4.9	3.4	3.5	68.2
4	6.0	7.5	6.0	6.5	40.9
5	1.8	4.3	3.0	3.0	72.9
Mean	5.3	7.7	6.2	6.4	52.3
* LSD*_0.05_ for: cultivars–ns; objects—1.9; interaction: cultivars × objects—ns					

Objects	Cultivars			Mean	Reduction (t ha^−1^ relative to control)
	Bartek	Gawin	Honorata		
Non-commercial yield of potato tubers in t ha^−1^					
1	3.25	3.52	5.68	4.15	–
2	3.12	3.50	3.21	3.27	0.88
3	2.65	2.21	2.51	2.46	1.69
4	3.10	3.19	2.77	3.02	1.13
5	1.41	1.29	1.58	1.43	2.72
Mean	2.70	2.74	3.15	2.86	1.61
* LSD*_0.05_ for: cultivars–ns; objects–2.28; interaction: cultivars × objects–ns					

*LSD* least significant difference; *ns* not significant (average over three years).

1—Control object; 2—Harrier 295 ZC; 3—Harrier 295 ZC + Kelpak SL; 4—Sencor 70 WG; 5—Sencor 70 WG + Asahi S.

**Table 6:** Number of weeds and non-commercial yield of potato in study years.

Years	Cultivars			Mean
	Bartek	Gawin	Honorata	
Number of weeds per 1 m^2^ before potato row closing				
2012	8.2	9.1	10.6	9.3
2013	4.8	5.9	7.1	5.9
2014	2.2	2.6	2.6	2.5
Mean	5.1	5.9	6.8	5.9
*LSD*_0.05_ for: cultivars—ns; years—1.2; interaction: cultivars × years—ns				
Number of weeds per 1 m^2^ before harvest of the potato				
2012	6.1	9.7	10.1	8.6
2013	5.1	8.1	4.7	6.0
2014	4.6	5.3	3.8	4.6
Mean	5.3	7.7	6.2	6.4
*LSD*_0.05_ for: cultivars—ns; years—1.3; interaction: cultivars × years—2.3				
Non-commercial yield of potato tubers in t ha^−1^				
2012	8.81	7.39	7.25	7.82
2013	5.03	3.29	2.03	3.45
2014	9.67	3.78	1.97	5.14
Mean	7.84	4.82	3.75	5.47
*LSD*_0.05_ for: cultivars–ns; years—1.31; interaction: cultivars × years—2.42				

*LSD* least significant difference, *ns* not significant.

The highest weed infestation at the beginning of potato plant vegetation, in all cultivars grown, was recorded on the mechanically tended control plot and averaged 14.3 weeds per square meter. Significantly fewer weeds were found on the other sites. On the other hand, the fewest weeds and the highest destruction efficiency were recorded on objects sprayed with herbicides and biostimulants (objects 3 and 5). Also, it was found^12^ that the least weed infestation and highest treatment efficiency was after application of Avatar 293 ZC herbicide and Avatar 293 ZC herbicide and GreenOK Universal—PRO biostimulant. The number of weeds determined before harvest also depended on the treatment and years of study (Tables 5 and 6). The number of weeds per square meter ranged from 3.0 to 11, and was lowest after application of Sencor 70 WG herbicide and Asahi SL biostimulant (object 5). Weed destruction efficiencies ranged from 27.2 to 72.9% and were also highest on object 5^14^ using herbicide Avatar 293 ZC + biostimulant PlonoStart and herbicide Avatar 293 ZC + biostimulant Agro-Sorb Folium achieved weed destruction efficiencies of 60.8 and 70.5%, respectively. High efficiency of weed destruction reaching 83% was shown^26^ At the same time, these authors^26^ found that the uptake of nutrients (N, P, K) by weeds was highest on sites with high weed infestation. In the dry year of 2012, the number of weeds in both determination dates was the highest, while in other years it was significantly lower. The influence of weather conditions on weed infestation and weed destruction efficiency is confirmed by the studies of other authors^12,14^. In the number of weeds determined before harvesting the tubers, no significant interactions between cultivars and the objects were found. However, significant interactions between cultivars and years of research were found (in the number of weeds marked before tuber harvesting). The biggest value was recorded in 2012 in the Honorata cultivar (10.1), and the lowest one, in the same cultivar in 2014 (3.8). This indicates a significant impact of rainfall and thermal conditions on the number of segetal plants on potato plantations.

### Yield structure and non-commercial yield

The percentage of tuber fraction in yield was differentiated by methods of care (Table 7).

**Table 7:** Effect of biostimulants with herbicides on the percentage and weight fraction of tubers (mean for 3 years and cultivars).

Objects	Potato tuber fractions in mm				
	˂35	36–50	50–60	˃60	˃36 to ˃60
1	11.5	28.0	31.9	28.6	88.5
2	7.9	27.9	37.2	27.0	92.1
3	5.1	28.3	31.6	35.0	94.9
4	6.3	24.6	37.3	31,8	93.7
5	3.2	29.4	34.5	32.9	96.8
Mean	6.8	27.6	34.5	31.1	93.2

On the control object, the largest number of small tubers with a diameter of less than 35 mm and the smallest number of commercial tubers with a diameter of more than 35 mm were determined. However, on the objects sprayed with herbicides and biostimulants, the percentage of small tuber weight was the lowest. The favorable effect of treatment with herbicides and biostimulants on the formation of potato yield structure is reported by other authors^10,11,27^. The share of small tubers in the yield was reflected in the formation of the yield of non-commercial tubers, to which the weight of tubers with defects was still added. The non-tradable yield depended significantly on the methods of care and weather conditions in the years of the study (Tables 5 and 6). The lowest non-commercial yield was harvested from the least weedy objects where herbicides and biostimulants were applied. The reduction in non-commercial yield on objects 2–5 was in the range of 0.88–2.72 t∙ ha^−1^. It was found that growth regulators Kelpak SL, Asahi SL increased the concentration of phenolic compounds in plants, which are involved in the defense mechanism against environmental stresses^5^. At the same time, these preparations increased the proportion of medium-sized tubers in the yield and caused a significant increase in tuber yield of the potato varieties studied. It was evaluated an organic biostimulant containing algae extracts that was applied to the plant leaves of potato cv. Sante and it was found that it improved plant growth parameters, including plant height, stem number, tuber yield and tuber quality (dry matter, protein, N, P and K content were higher)^28^. The usage the biostimulants Kelpak SL (Ecklonia maxima) and HumiPlant (fulvic acids from leonardite) made it possible to observe that they increased plant assimilative area, abiotic stress tolerance, marketable tuber yield, reducing non-marketable yield, and thus increased the marketability of cultivars^29^. The statistical analysis showed no significant interactions between cultivars and objects and the on non-commercial yield of the studied potato cultivars. Analyzing weather conditions during potato vegetation showed that the lowest non-commercial yield of tubers was harvested in 2013, which was the optimal season in terms of moisture and temperature (Tables 4 and 6). The similar observations were found by using Bio-algeen S90 and Keplak SL containing seaweed extracts yielded better production results in the warm and very wet growing season^29^. The optimal weather conditions that are conducive to good potato yields are an average May-September air temperature of 15.2 °C and a rainfall of 340–400 mm^23^. The studied cultivars did not significantly affected on non-commercial tuber yield. However, the interactions between cultivars and weather conditions during the years of research have been proved. This means that the cultivars responded differently to weather conditions during the growing seasons. The lowest non-commercial yield was recorded for the Honorata cultivar in 2014 (1.97 t ha^−1^). The correlation analysis carried out showed a significant positive relationship between the non-commercial yield of potato and the number of weeds and the air-dry weight of weeds determined before short-circuiting the rows and before harvesting the tubers, which confirms that the higher the number and weight of weeds per 1 m^2^, the higher the non-commercial yield of potato (Table 8).

**Table 8:** Correlation coefficients between weed number and air-dry weight of weeds and non-commercial yield of potato (mean for 3 years and cultivars).

Elements of weed infestation	Non-commercial yield of potato tubers in t ha^−1^
Number of weeds per 1 m^2^ before row closing	+ 0.9665
Number of weeds per 1 m^2^ before harvest of the potato	+ 0.9215
Air–dry weight of weeds in g m^−2^ before row closing	+ 0.9106
Air–dry weight of weeds in g m^−2^ before harvest of the potato	+ 0.9750

It was noted a significant negative correlation between the number and fresh weight of weeds and trade fraction tuber yield and yield of large potato tuber (correlation coefficients ranged from − 0.9269 to − 0.9798)^14^^.^ Also, it was found a strong negative linear correlation (r = − 0.90) between the presence of weed species and total yield of potato (t ha^−1^), which means that a decrease in the number of weeds caused an increase in total yield^30^.

## Conclusions

1. The herbicides and biostimulants used in the experiment had a significant effect on reducing the number of weeds occurring at the beginning and end of potato vegetation and on the size of the non-commercial yield of tubers.
2. The least weeds and the best herbicidal effect were obtained after application of herbicide Sencor 70 WG + biostimulant Asahi SL and herbicide Harrier 295 ZC + biostimulant Kelpak SL.
3. Effective reduction of weeds resulted in improved yield structure and reduced non-commercial yield of potato.
4. Weather conditions in the years of the study significantly determined the number of weeds and non-commercial yield of potato. The lowest non-commercial yield was harvested in 2013, when moisture-thermal conditions were optimal.
5. The studied cultivars Bartek, Gawin and Honorata, belonging to the same earliness group (medium early), had no significant impact on the number of weeds determined before row closing and tuber harvesting and on the non-commercial potato yield.

## Data Availability

The datasets generated and analysed during the current study are not publicly available due to they are the authors' own data, but are available from the corresponding author on reasonable request.
